# Assessment of Craniofacial Growth Pattern Relative to Respiratory Mandibular Movement and Sleep Characteristics: A Pilot Study

**DOI:** 10.1055/s-0044-1795120

**Published:** 2024-12-30

**Authors:** Sukaynah Al-Awami, William Tanberg, Alberto Monegro, David Covell Jr, Jean-Benoit Martinot, Thikriat Al-Jewair

**Affiliations:** 1 Department of Orthodontics, School of Dental Medicine, State University of New York at Buffalo, Buffalo, New York, United States; 2 Pediatric Sleep Center, School of Medicine, University at Buffalo, Buffalo, New York, United States; 3 Sleep Laboratory, CHU Université Catholique de Louvain (UCL) Namur Site Sainte-Elisabeth, Institute of Experimental and Clinical Research, UCL Bruxelles Woluwe, Brussels, Belgium

**Keywords:** sleep, mandibular movements, craniofacial pattern

## Abstract

**Objectives** The primary objective was to evaluate the influence of sagittal skeletal pattern on mandibular movement (MM) during sleep in growing orthodontic populations. The secondary objective was to compare MM according to obstructive sleep apnea (OSA) status.

**Materials and Methods** This cross-sectional study included subjects between 6 and 17 years old, presenting with class I, II, and III skeletal patterns and no previous history of orthodontic treatment. A wireless sensor connected to the patient's chin before bedtime and removed the next day was used to record MM signals. The signals were analyzed using a machine learning algorithm to measure sleep and MM outcomes. MM variables included percentage change in waveform prominence (%), variance in peak prominence, mean prominence values, length of events (seconds), respiratory rate per minute, dominant frequency, and amplitude of dominant frequency. The obstructive respiratory disturbance index determined from the sensor was used to confirm OSA status.

**Results** There was no statistically significant difference in MM variables between class I, II, and III subjects. When compared according to OSA status, the amplitude of dominant frequency was significantly higher in the OSA than the non-OSA group ( *p* = 0.005). When evaluated according to both skeletal classification and OSA status, the class I OSA subjects showed a higher median value than the non-OSA class I group ( *p* = 0.016).

**Conclusion** Within the limits of this study, the sagittal skeletal pattern had no effect on the respiratory MM. This study did not find a correlation between craniofacial pattern and MM and OSA.

## Introduction

Over the last two decades, sleep deprivation has increased among children, adversely affecting their physical, mental, and psychological health. 1 One of the factors contributing to this epidemic is sleep-disordered breathing (SDB)—specifically, obstructive sleep apnea (OSA). 1 OSA is characterized by repeated episodes of prolonged or intermittent upper airway obstruction, partial or complete, that disrupts pulmonary ventilation, oxygenation, and normal sleep patterns. 1 2 3

In the United States, 1 to 4% of children suffer from OSA. 3 4 If left untreated, OSA may result in several negative consequences, such as daytime sleepiness, behavioral and neurocognitive deficiency, and growth and developmental delay. 1 3 4 It was also suggested that children with severe OSA may be at increased risk of chronic diseases later in life. 5

According to recent studies, mandibular movement (MM) during sleep is correlated with sleep and breathing 6 7 and plays a role in the pathophysiology of OSA. 8 9 During sleep, the mandibular jaw opens, narrowing the upper airway dimension and increasing upper airway collapsibility. 8 While jaw opening increases with deepening sleep in normal subjects, OSA patients showed an even more increased jaw opening in all sleep stages than patients without OSA. 9 10 In addition, jaw opening in OSA patients was found to be greater than that of normal patients at both the end of expiration and the end of inspiration during sleep. 9 The greater jaw opening results from the functional abnormalities of the upper airway dilating muscles, mainly the genioglossus. 11 12 The importance of this muscle in the maintenance of upper airway patency is well established. 13

MM assessment is a sensitive and specific approach that allows the MM signal to be read visually to assess sleep–wake state and respiratory events. 14 Martinot et al 15 assessed the degree of agreement between MM and the gold standard diaphragmatic electromyography (EMG-d) signals during different sleep stages and abnormal respiratory events. They concluded that the changes in amplitude of respiratory MM during sleep closely parallel the variations in diaphragmatic EMG-d activity and allow for accurate identification of respiratory effort (RE) and respiratory drive. Monitoring the MM signals provides useful information about the obstructive nature of SDB and rapid eye movement (REM) sleep changes in ventilatory drive. 15

MMs can be analyzed according to their amplitude and duration. 7 In one study, the MM signal was segmented using a continuous wavelet transform to identify sudden movements of high amplitude. Peak-to-peak amplitude of MM excursion during a respiratory cycle, inspiration, and expiration, was measured by grayscale mathematical morphology. 7 Assessments of MM with a high-resolution magnetometer can accurately identify cortical arousals, RE, and RE-related arousals (RERAs). Additionally, analysis of MM can precisely approximate sleep duration and detect mouth opening as an indicator of oral breathing. Importantly, because MMs are not influenced by head position, the MM signal can be reliably and consistently detected throughout sleep. 6

The association between respiratory MM, a proxy for OSA, and craniofacial growth pattern has not been investigated. This study aims to address the limitations of previous literature and assess for the first time the association between respiratory MM and craniofacial growth pattern. The primary objective of this study was to evaluate the influence of sagittal skeletal patterns on respiratory MM during sleep in growing orthodontics patients. The secondary objective was to compare MM between OSA and non-OSA patients. It was hypothesized that the craniofacial growth pattern has an influence on MM parameters when compared according to anteroposterior skeletal classification, and there is a significant difference in the pattern of MM between OSA and non-OSA patients.

## Materials and Methods

### Study Design

This cross-sectional study compared respiratory MM during sleep in growing orthodontic patients presenting with class I, II, and III skeletal patterns. The data were collected at the University at Buffalo School of Dental Medicine's orthodontic clinic. The study was approved by the University at Buffalo Health Sciences Institutional Research Board (#00004693) and was conducted between June 2021 and April 2022.

### Same Size Estimation

A minimum sample of 48 subjects (8 per each of the six groups composed of the three skeletal classifications and two OSA statuses) was considered adequate to detect an effect size of 0.8 (Cohen's *d* ) among the groups at a power of 80% and 5% type I error rate.

### Sample Selection

Subjects presenting for orthodontic treatment were screened for inclusion according to preset criteria. Inclusion criteria were children and adolescents between 6 and 17 years old, presenting with skeletal class I, II, and III malocclusion according to ANB (°) 16 and Wits appraisal (mm). 17 18 19 Subjects were excluded if they had temporomandibular disorders, history of trauma to the face or jaws, congenital or acquired craniofacial deformities, history of adenotonsillectomy, history of orthodontic treatment, and current or past bruxism diagnosis.

Eligible subjects were invited to participate at their orthodontic screening visit. Written informed consent/assent was obtained from patients and their parents/guardians. Initial records including lateral cephalogram images were obtained for all subjects. The cephalograms were digitally traced using Dolphin Imaging Software (version 11.95) then subjects were divided into three groups: class I malocclusion (ANB° 1–4; Wits −1.8 to 0.8 mm), class II malocclusion (ANB° >4; Wits >0.8 mm), and class III malocclusion (ANB° <1; Wits < − 1.8 mm). All data were obtained and measured by one investigator (S.A.).

### Study Procedures

The MM waveform was recorded using an inertial measurement sensor (Sunrise, Erpent, Belgium, FDA# 21 CFR 868.2376). For this home-based sleep study, subjects were given a coin-sized sensor and were instructed to adhere the sensor to the chin in the mentolabial sulcus before bedtime and remove it the next morning for a convenient home-based sleep study. The sensor communicated with a smart phone application via Bluetooth technology. The collected MM data were then automatically transferred to a cloud-based infrastructure. As described below, MM signal components were analyzed using a machine learning algorithm to automatically detect wake, arousal, RE and respiratory disturbances, and quiet sleep.

**Fig. 1:**
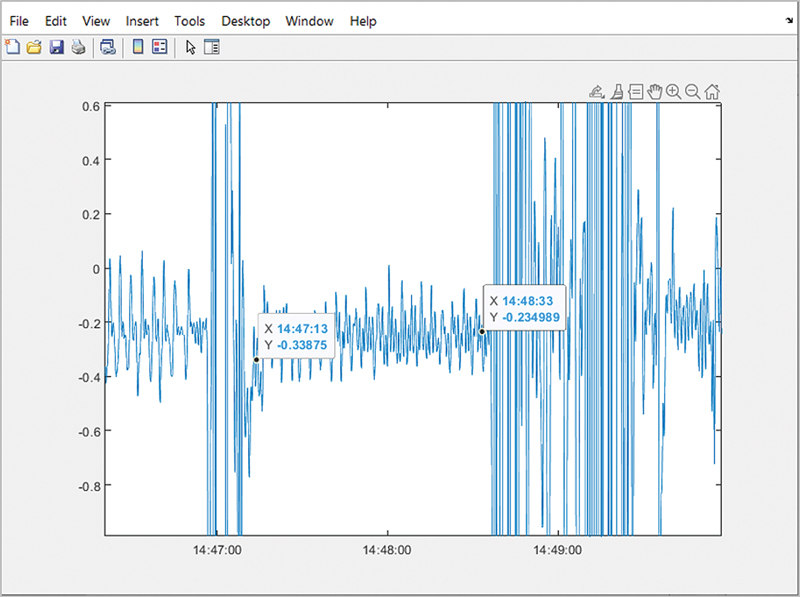
Enveloped data with the event start and stop times inputted.

For data analysis, the longest obstructive event during a sleep night was identified for each patient and the pattern of MM during the event was then analyzed as follows. The MM raw data were transferred in European Data Format (edf) format to MATLAB (software: MATLAB R2022a, add-ons: Signal Processing Toolbox [version 9.0], edfRead [version 2.10.0.1]) utilizing the edfRead add-on. There were six channels recorded for MM signals, the first three recorded linear speed, while the last three recorded angular speed (rotational velocity). For this study, only angular speed channels were used to identify the longest obstructive event. Data from each channel was stored and formatted to the correct start time before being viewed with Signal Analyzer. The data were then visually searched for the characteristic patterns of the event. Any abnormalities or excessive noise that may disqualify one of the channel choices was noted at this point. Channel signals were then enveloped in Signal Analyzer (Pre/Post_Envelope) and exported back into the MATLAB workspace. The same event was located on a MATLAB plot to identify the start and end time of the event (hours:minutes:seconds), since the time in Signal Analyzer was in decimal hours. The remainder of the script was run using the enveloped data with the event start and stop times inputted ( Fig. 1 ). The choice of which angular speed channel data to use was based on the channel with the greatest variance. All analyses were completed by one investigator (W.T.) and confirmed by a sleep physician (A.M.).

**Fig. 2:**
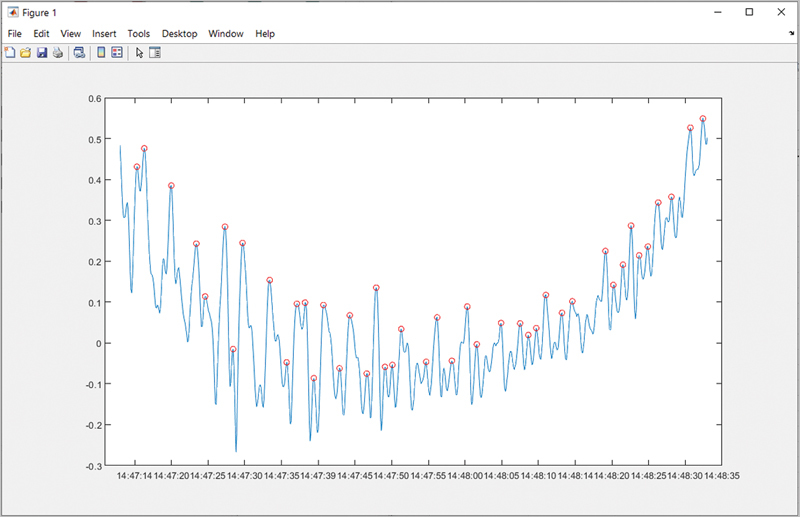
Enveloped event peaks marked as red dots.

The primary outcome, MM pattern during the longest obstructive event, was measured using seven variables:

- Length of an obstructive event (seconds): length of events in seconds within a total sleep night.
- Respiratory rate per minute (rate/min): calculated as number of peaks/length of events to give an estimate of respiratory rate.
- Mean prominence: the prominence of a peak measures the peak's height and location in relation to other peaks. 20 It is calculated as mean peaks of event/mean peaks of the entire sleep study of the same individual ( Fig. 2 ).
- Percentage change in peak prominence (%): calculated as Max(prominence) − Min(prominence)/Max(prominence).
- Variance of prominence: peak prominence to peak prominence variability in amplitude of the angular change measured in degrees per second.
- Dominant frequency: measures the frequency with the most energy. Fast Fourier transform (FFT) was used to determine the dominant frequency in that event. FFT is a mathematical signal analysis method that transforms the signal from a time presentation to a frequency presentation and thus allowing its calculation and measurement.
- Amplitude of dominant frequency (power density): the maximum extent of a vibration or oscillation, measured from the position of equilibrium.

The secondary outcomes included objective sleep variables obtained from the sensor and subjective measures obtained from the Pediatric Sleep Questionnaire (PSQ). 21 22 The objective measures included total sleep time (TST; hour:minute), apnea–hypopnea index (AHI; events/hour), sleep efficiency (SE; %), REM sleep (% of TST), arousal index (events/hour), obstructive respiratory disturbance index (ORDI; events/hour), ORDI _supine_ (events/hour), ORDI _nonsupine_ (events/hour), ORDI _REM_ (events/hour), ORDI _non-REM_ (events/hour), RE (% of TST), RERA (number/h).

Presence or absence of OSA was determined based on the ORDI (events/hour). A score greater than or equal to 5 indicated the presence of OSA. 23 Those with OSA were referred to the UB Jacobs School of Medicine and Biomedical Sciences pediatric sleep clinic for further evaluation. To ensure appropriate recording and mitigate any sensor malfunctions, the MM studies were repeated at two consecutive nights, only the night with a highest ORDI was included in the analysis.

The PSQ was filled out by parents/guardians during the initial records or the consultation appointments. It consisted of 22 questions that asked about snoring, breathing difficulty during sleep, hyperactive or inattentive behavior, daytime sleepiness, and other pediatric OSA items. Eight or more answers of “yes” was considered high risk of OSA. 21 In addition, demographic variables including chronological age, skeletal age utilizing the cervical vertebral maturation method, sex, body mass index (BMI), and neck circumference were recorded for all subjects.

### Statistical Analysis

Data normality was assessed using the Shapiro–Wilk test. The Kruskal–Wallis test was used in the comparison of MM variables between skeletal classification groups. To compare MM according to OSA status, the Mann–Whitney rank sum test was used due to normality assumption violations in at least one of the groups, or the Welch's *t* -test when both groups fail to reject Shapiro–Wilk test for normality.

Post-hoc testing was performed on the two sleep variables (SE and TST) using Dunn tests with Bonferroni adjustment for pairwise analysis. In the case of the amplitude of dominant frequency, two linear models were constructed on the log transform of that variable. The first model was a simple linear model with the covariate of OSA group. The second model included both OSA group and skeletal classification. Additionally, a post-hoc power analysis was performed using the OSA and non-OSA sample data to estimate an effect size.

## Results

Forty-five subjects were included in this study ( Table 1 ). Forty-two percent ( *n* = 19) had class I skeletal pattern, 38% ( *n* = 17) had class II, and 20% ( *n* = 7) had class III. There were no significant differences between the skeletal classification categories in terms of age and sex. The BMI ranged from 13.5 (underweight) to 32.6 (overweight); however, there was no significant difference in the median BMI among the three skeletal groups.

**Table 1:** Sample characteristics

Variable	Class I, *N* = 19	Class II, *N* = 17	Class III, *N* = 9	Total, *N* = 45	*p* -Value
Age (y)					
Median (IQR)	13 (2.5)	12 (2)	14 (3)	13 (2)	0.688 a
Range	7–16	9–17	9–14		
Sex, *N* (%)					
Female	12 (27)	11 (24)	3 (7)	26 (58)	0.282 b
Male	7 (16)	6 (13)	6 (13)	19 (42)	
CVM stage, *N* (%)					
Prepubertal	4 (9)	1 (2)	3 (7)	8 (18)	0.117 b
Pubertal	6 (13)	10 (22)	1 (2)	17 (37)	
Postpubertal	9 (21)	6 (13)	5 (11)	20 (45)	
SNA (°)					
Median (IQR)	83.9 (4.9)	83.2 (3.8)	81.6 (5.12)	82.9 (4.6)	0.264 a
Range	78.8–87.12	76.2–85.2	75.1–83.92	75.1–87.12	
SNB (°)					
Median (IQR)	81 (6.38)	78 (3)	82.7 (7.73)	80.6 (5.7)	0.024 a
Range	75.3–85.12	70.2–79.1	76.6–85.23	70.2–85.23	
ANB (°)					
Median (IQR)	2.5 (1.35)	5.8 (0.9)	−0.4 (1.23)	3.3 (3.8)	<0.001 a
Range	1.4–3.35	4.3–6	−4.7–0.1		
Sn-GoGn (°)					
Median (IQR)	25.5 (6.75)	29.2 (6.3)	22.6 (13.12)	26.3 (8.9)	0.080 a
Range	19.7–30.3	22.4–32.6	14.9–29.75		
BMI					
Median (IQR)	20.2 (3.75)	21.2 (4.7)	20.9 (2.98)	20.7 (4.7)	0.704 a
Range	15–29	13.5–29.3	15.3–32.6	13.5–32.6	
Neck circumference (cm)					
Median (IQR)	32.5 (4.5)	32 (4)	33 (4.5)	32 (4)	0.322 a
Range	20–37	27–38	26–38	20–38	

Abbreviations: CVM, cervical vertebral maturation; IQR, interquartile range.

a Kruskal–Wallis test, significance set at 5%.

b Fisher's exact test, significance set at 5%.

When compared according to skeletal classification, the Kruskal–Wallis test showed no statistically significant difference in the median of all measured MM variables ( Table 2 ). It is worth noting however that the variance in peak prominence and mean prominence values were smallest among the class II subjects in the sample. These differences were not statistically significant at the 5% level.

**Table 2:** Mandibular movement pattern according to skeletal classification

	Class I		Class II		Class III		*p* -Value a
Variable	Median	IQR	Median	IQR	Median	IQR	
Length of event (s)	157.1	108.75	171.10	60.00	223.10	121.00	0.180
Respiratory rate/minute	20.30	8.10	20.46	6.99	29.31	8.29	0.357
Mean prominence values/mean entire study	0.75	0.53	0.36	0.27	0.61	0.54	0.083
% Change in prominence	75.62	9.56	76.32	17.86	82.15	7.85	0.207
Variance of prominence	0.05	0.11	0.02	0.02	0.04	0.08	0.096
Dominant frequency (FFT)	0.53	0.20	0.19	0.48	0.53	0.53	0.496
Amplitude of dominant frequency (FFT)	0.10	0.07	0.08	0.08	0.07	0.05	0.839

Abbreviations: FFT, fast Fourier transform; IQR, interquartile range.

a Kruskal–Wallis test, significance set at 5%.

**Fig. 3:**
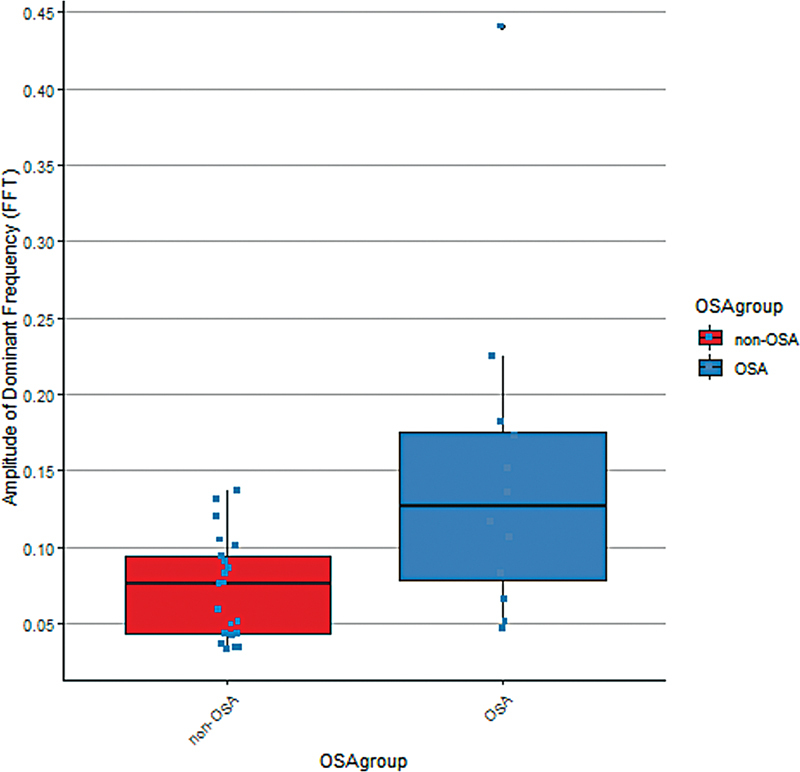
Boxplots of amplitude of dominant frequency according to OSA status. OSA, obstructive sleep apnea.

The MM was also analyzed according to OSA status ( Table 3 and Fig. 3 ). A significant difference in distribution between OSA and non-OSA was found in amplitude of dominant frequency ( *p* = 0.005). Using a log transformation on the amplitude of dominant frequency, and linearly modeling it versus the OSA status resulted in a significant model. The model showed an average increase in the log of amplitude of dominant frequency of 0.62 ( *p* = 0.004) from non-OSA to the OSA. This would be an average increase in amplitude of dominant frequency from non-OSA to OSA by a factor of 1.86. To account for skeletal pattern, a second model including skeletal classification as a covariate with interactions was constructed ( Table 4 ). While the model indicated a significant difference in the log of amplitude of dominant frequency for class I between OSA and non-OSA groups (−0.84, *p* = 0.020), the overall model itself was not significant ( *F-* test, *p* = 0.067).

**Table 3:** Mandibular movement pattern according to OSA status (ORDI ≥ 5)

	OSA		Non-OSA		*p* -Value a
Variable	Median	IQR	Median	IQR	
Length of event (s)	176.60	134.75	177.10	90.00	0.985
Respiratory rate/minute	22.78	11.33	20.46	10.57	1
Mean prominence values/mean entire study	0.46	0.59	0.57	0.48	0.618
% Change in prominence	80.07	13.01	76.37	15.50	0.754
Variance of prominence	0.04	0.15	0.03	0.05	0.754
Dominant frequency (FFT)	0.47	0.54	0.51	0.41	0.754
Amplitude of dominant frequency (FFT)	0.13	0.10	0.08	0.05	0.005

Abbreviations: FFT, fast Fourier transform; OSA, obstructive sleep apnea.

a Mann–Whitney rank sum test, significance set at 5%.

**Table 4:** Amplitude of dominant frequency (FFT) according to skeletal classification and OSA status

Group	Skeletal classification	Estimate	SE	df	t.ratio	*p* -Value a
non-OSA–OSA	I	−0.84	0.34	27.00	−2.46	**0.020**
non-OSA–OSA	II	−0.43	0.34	27.00	−1.27	0.214
non-OSA–OSA	III	−0.75	0.39	27.00	−1.90	0.069

Note: Statistically significant *p* -value is indicated in bold.

a Estimated marginal means between non-OSA and OSA within skeletal classification using log(amplitude of dominant frequency), significance set at 5%.

Overall, 36% of subjects had an ORDI of ≥5, which is suggestive of OSA, although no significant difference in OSA diagnosis was found between the three skeletal pattern groups. See Table 5 for comparisons of objective sleep variables among class I, II, and IIII skeletal patterns. The median SE in class III skeletal pattern was 77.5% compared with 84% in class II and 84% in class I where the difference was of borderline significance, *p* = 0.049. Also, the median TST was significantly higher in skeletal class II in comparison to class I and III ( *p* = 0.034). Dunn's test was performed on these two variables to examine the pairwise relationships. Using Bonferroni adjustment, a significant difference was found between the class I and class III groups for the SE variable ( *p* = 0.044). None of the other variables showed statistical differences.

**Table 5:** Comparison of sleep variables between skeletal class I, II, and III malocclusion

Variable	Class I		Class II		Class III		
	Median	IQR	Median	IQR	Median	IQR	*p* -Value a
AHI	2.55	0.50	2.20	0.80	2.05	0.52	0.148
SE %	84.00	2.25	84.00	8.00	77.50	8.00	**0.049**
TST (h)	6.62	2.74	7.55	1.25	6.38	2.50	**0.034**
REM sleep (% of TST)	8.50	8.75	8.00	9.00	8.50	2.25	0.927
AI (events/h)	18.10	4.78	17.40	6.70	21.35	5.60	0.719
ORDI _All_ (events/h)	3.90	2.65	3.90	2.40	4.90	3.48	0.570
ORDI _Supine_	1.15	3.52	0.00	1.30	2.55	5.52	0.268
ORDI _nonsupine_	4.00	2.83	3.90	2.70	4.65	3.65	0.542
ORDI _REM_	4.80	6.67	1.30	6.00	2.65	5.07	0.429
ORDI _non-REM_	3.55	2.88	2.90	2.30	3.15	3.12	0.893
RE (% of TST)	18.00	8.75	17.00	8.00	21.00	6.75	0.321
RERA (events/h)	0.75	0.65	1.00	1.00	1.45	1.23	0.280

Abbreviations: AHI, apnea hypopnea index; AI, arousal index; IQR, interquartile range; ORDIAll, obstructive respiratory disturbance index-all events; ORDInon-REM, obstructive respiratory disturbance index during non-REM sleep; ORDInon-supine, obstructive respiratory disturbance index in the nonsupine position; ORDIREM, obstructive respiratory disturbance index during REM sleep; ORDISupine, obstructive respiratory disturbance index in the supine position; REM, rapid eye movement; SE, sleep efficiency; TST, total sleep time.

Note: Statistically significant *p* -values are indicated in bold.

a Kruskal–Wallis test, significance set at 5%.

Results from the PSQ indicated a significant difference in the average “Yes” counts between the OSA and non-OSA groups ( *p* = 0.003; Table 6 ). However, there were no significant differences between skeletal class I, II, and III groups.

**Table 6:** Risk for OSA determined from the PSQ according to skeletal classification and OSA status

Variable		Mean	SD	Median	IQR	*p* -Value
**Skeletal classification**	Class I	3.58	2.63	4.00	3.00	0.107 a
	Class II	4.71	2.89	4.00	5.00	
	Class III	2.56	3.43	1.00	2.00	
**OSA status (ORDI ≥ 5)**	Non-OSA	3.13	2.28	3.00	3.00	**0.003** b
	OSA	8.17	3.19	9.00	1.50	

Abbreviations: ORDI, obstructive respiratory disturbance index; OSA, obstructive sleep apnea.

a Kruskal–Wallis test, significance set at 5%.

b Mann–Whitney U-test, significance set at 5%.

A post-hoc power analysis was performed under the assumptions of a two-sided *t* -test, and a Cohen's *d* effect size of 0.95. This effect size was obtained using the sample means and standard deviations of amplitude of dominant frequency for the OSA and non-OSA groups. Using the smallest group size ( *n* = 12) results in a power of 60.2%. To obtain a power of 80 and 90%, group sample sizes would need to be 19 and 25, respectively.

## Discussion

Since children with SDB show a characteristic facial appearance (retrognathic mandible, a short nasal floor, increased anterior lower facial height or posterior facial height, increased interincisal angle with retroclined mandibular incisors, a narrow pharyngeal airway space, an anterior tongue base position, and a long soft palate) at an early age 24 and since MM pattern closely represents sleep and breathing, this study hypothesized that the craniofacial pattern could influence the respiratory MM in growing patients presenting for orthodontic treatment. Our findings suggested that there was no significant difference in MM for any of the investigated variables when the three skeletal classifications were compared, thus the hypothesis was rejected. This could be attributed to several factors such as the severity of the craniofacial pattern of the included sample where ANB angle medians and ranges showed that the sample included only mild class II and III skeletal pattens. This may have resulted in a regression to the mean when the comparisons were investigated. The lack of a difference among skeletal patterns could also be attributed to the small sample size.

MM was also compared between OSA and non-OSA subjects. The amplitude of dominant frequency (frequency with the largest amplitude) was significantly different between the two groups in skeletal class I subjects with the OSA group showing a higher mean value ( *p* = 0.020). Martinot et al 6 explained that OSA patients presented with higher amplitude frequency in MM as compared with healthy controls. While the model suggested a significant difference within the classes, these findings need to be interpreted with caution due to the small sample size. Future larger studies are warranted for proper analysis and inferences.

Detection of MM pattern is a valid method to identify RE associated with OSA and microarousals. 6 The main goal for OSA treatment is normalization of RE, which could be assessed more easily and accurately by observing mandibular position during sleep. 7 This reliable and simple surrogate measure of RE and indirectly of arousal could be a more accessible alternative to other invasive methods such as sleep laboratory obtained polysomnography (PSG). 7 Martinot et al 15 reported good agreement between diaphragmatic activity objectively measured via EMG-d and MM when evaluating RE in patients with SDB. For each 10 μV increase in EMG-d activity, MM amplitude increased by 0.28 mm. This association remained valid even during REM sleep, during which EMG-d and MM amplitudes were reduced. They concluded that changes in amplitude of respiratory MM during sleep closely paralleled the variations in EMG-d activity and permit for accurate identification of RE and respiratory drive. 15

In this study, OSA diagnosis was based on ORDI obtained from the sensor and supplemented with the PSQ. It is possible in this study that some patients had false-negative results due to the automated diagnosis which does not always take into account differences related to genetic make-up or ethnicity where automation had shown limitations 25 or internight variability in sleep measurement. 26 Using sleep laboratory PSG remains the gold standard method to diagnose OSA.

Saleh 27 analyzed MM in growing patients pre- and post-rapid maxillary expansion (RME) and found no significant differences in percent (%) change or event variance, but there was a significant short-term decrease in obstructive event duration and frequency of MM peaks after RME treatment in children and adolescents. However, the authors did not assess the amplitude of dominant frequency and thus comparisons with previous studies are not possible.

DelRosso et al 28 analyzed the obstructive apnea and hypopnea duration in children undergoing PSG for the evaluation of snoring and found the apnea/hypopnea event duration to be 7 to 8 seconds in the youngest children and 10 seconds above the age of 6 years. They concluded that apnea and hypopnea length increase with age in children and adolescents without association with gender or OSA severity. Butler et al 29 reported that the event duration in adults varied from 11.2 to 57.6 seconds (mean: 21.3 seconds; median: 20.6 seconds). Subjects with shorter event durations were more likely to be younger, female, African American, and current smokers. A shorter event was also associated with higher BMI, lower AHI, and higher minimum blood oxygen saturation. Event duration was not significantly correlated with TST or SE. They suggested that event duration predicted mortality beyond AHI and stated that the predictive information associated with event duration was stronger in individuals with moderate to severe OSA. In this study, OSA was of a mild severity.

In the current study, the median length of event was 157.1 seconds, 171.1 seconds, 223.1 seconds in skeletal class I, II and III, respectively. It showed a longer event duration in class III, but the differences were not statistically significant. The findings of this study showed a quite long event duration range. The reasoning behind this presentation is that these events include RERAs in addition to the obstructive apnea or hypopnea. Also, this study did not account for maxillary transverse width or intraoral soft tissue characteristics such as Mallampati classification which might have contributed to the lack of significance between the groups.

Galeotti et al 3 30 found a significant correlation between maxillomandibular discrepancy and OSA severity. They stated that the reduction of nasopharyngeal width was correlated with maxillomandibular hyperdivergent growth pattern. They explained that severe OSA condition can result in increased mouth opening and mouth breathing. That in turn has a great effect on growing children and can lead to a hyperdivergent skeletal pattern. 30 In the current study, cephalometric variable measurements showed a normodivergent facial pattern, normal lower facial height, and the horizontal and the vertical growth were well-balanced according to the *Y* -Axis. Both OSA and non-OSA showed no significant difference in any of the measurements.

This study also evaluated the sleep variables in each skeletal classification group. The median SE in class III skeletal pattern was 77.5% compared with 84% in class II and 84% in class I and the difference was on borderline significance, *p* = 0.049. Also, the median TST was higher in skeletal class II in comparison to class I and III and the difference was statistically significant, *p* = 0.034. None of the other variables analyzed showed statistical differences among the three groups. This is similar to Saleh, 27 who found no significant differences in sleep variables following RME treatment in 14 pediatric patients with the exception of SE, which showed a significant increase from *T*_1_ (pre-expansion) to *T*_2_ (immediately post-expansion). Ashok et al 31 performed PSG testing on children who presented with high narrow palate, narrow maxillary arch, unilateral or bilateral posterior crossbite, class III malocclusion with anterior crossbite, signs and symptoms of apnea, and restless sleep as witnessed by parents. The PSG was obtained before maxillary expansion, after expansion, and 3 months of retention. They found an increase in the mean SE; however, the differences were not significant, but they found a significant improvement in TST.

### Study Limitations

Several limitations existed in the current study. The sample size was small in each skeletal classification group, which could affect generalizability of the results. Furthermore, due to the intensive labor associated with manually identifying an obstructive event and scoring and analyzing MMs, this study analyzed MM using only one event during a night sleep. The event was selected based on duration where it had to be of the longest duration compared with other events happening during sleep. Selecting multiple events and reporting averages might have resulted in different outcomes for MM associations with craniofacial pattern. Future studies are eagerly anticipated to explore this unique area of research.

## Conclusion

Within the limits of this study, the sagittal skeletal pattern had no effect on the respiratory MM. This study did not find a correlation between craniofacial pattern and MM and OSA.
